# Oxidative-Nitrosative Stress and Myocardial Dysfunctions in Sepsis: Evidence from the Literature and Postmortem Observations

**DOI:** 10.1155/2016/3423450

**Published:** 2016-05-04

**Authors:** M. Neri, I. Riezzo, C. Pomara, S. Schiavone, E. Turillazzi

**Affiliations:** ^1^Institute of Forensic Pathology, Department of Clinical and Experimental Medicine, University of Foggia, Ospedale Colonnello D'Avanzo, Viale degli Aviatori 1, 71100 Foggia, Italy; ^2^Institute of Pharmacology, Department of Clinical and Experimental Medicine, University of Foggia, Via L. Pinto 1, 71100 Foggia, Italy

## Abstract

*Background*. Myocardial depression in sepsis is common, and it is associated with higher mortality. In recent years, the hypothesis that the myocardial dysfunction during sepsis could be mediated by ischemia related to decreased coronary blood flow waned and a complex mechanism was invoked to explain cardiac dysfunction in sepsis. Oxidative stress unbalance is thought to play a critical role in the pathogenesis of cardiac impairment in septic patients.* Aim*. In this paper, we review the current literature regarding the pathophysiology of cardiac dysfunction in sepsis, focusing on the possible role of oxidative-nitrosative stress unbalance and mitochondria dysfunction. We discuss these mechanisms within the broad scenario of cardiac involvement in sepsis.* Conclusions*. Findings from the current literature broaden our understanding of the role of oxidative and nitrosative stress unbalance in the pathophysiology of cardiac dysfunction in sepsis, thus contributing to the establishment of a relationship between these settings and the occurrence of oxidative stress. The complex pathogenesis of septic cardiac failure may explain why, despite the therapeutic strategies, sepsis remains a big clinical challenge for effectively managing the disease to minimize mortality, leading to consideration of the potential therapeutic effects of antioxidant agents.

## 1. Introduction

Myocardial depression in the setting of sepsis and septic shock is common and has been recognized for a long time [1–5]. The presence of myocardial dysfunction in sepsis is associated with higher mortality. It has been shown that cardiovascular involvement increased mortality from 70% to 90%, compared to 20% in septic patients without myocardial impairment [2, 6]. Thus, cardiac dysfunction in sepsis is thought to have bad prognostic value as it coincides with its severity [6]. Septic cardiomyopathy is characterized by reversible biventricular dilatation, decreased ejection fraction, and impaired response to fluid resuscitation and catecholamine stimulation. However, septic myocardial impairment remains a clinical enigma [7, 8], and even its real incidence is uncertain due to the imprecision with which it is clinically described, the heterogeneity in patient selection in the published studies, and, finally, the lack of universally accepted definition of septic myocardial depression [9]. A reduced left ventricular ejection fraction (LVEF) is often used; on the other hand, septic cardiomyopathy can be defined as a global (systolic and diastolic) but reversible dysfunction of both the left and right sides of the heart [7, 10].

In recent years, the hypothesis that the myocardial dysfunction during sepsis could be mediated by ischemia related to decreased coronary blood flow waned and a complex mechanism was invoked to explain cardiac dysfunction in sepsis [11, 12].

In fact, septic shock is characterized by circulatory compromise, microcirculatory alterations, and mitochondrial damage, which all reduce cellular energy production. In order to reduce the risk of major cell death and a diminished likelihood of recovery, adaptive changes appear to be activated in sepsis. As a result, cells and organs may survive in a nonfunctioning hibernation-like condition. Sepsis-induced cardiac dysfunction may represent an example of such functional shutdown [13]. Furthermore, the effects of the host's immune-inflammatory response with particular focus on depressant molecules (i.e., TNF-alfa, IL-1), complement molecules, cellular adhesion molecules, and altered intracellular energetic and dysregulated intracellular calcium fluxes have been called upon in the pathophysiology of myocardial depression in sepsis [14–19]. A role of unbalance in oxidative status leading to high production of reactive oxygen species (ROS) has been hypothesized as playing a pivotal role in myocardial depression in sepsis. Nitric oxide (NO), a mediator involved in sepsis, is known to have a strong multifaceted influence on cardiac function since it affects the systemic and cardiac vascular tone and has direct effects on cardiomyocytes [20]. Finally, in the recent years, mitochondrial dysfunction has been considered as a crucial mechanism of heart impairment in sepsis [21]. Conclusively, a very complex pathogenesis involving a combination of hemodynamic, molecular, genetic, and metabolic cardiac alterations underlies cardiac involvement in sepsis.

In this paper, we review the current literature regarding the pathophysiology of cardiac dysfunction in sepsis focusing on the possible role of oxidative-nitrosative stress unbalance and mitochondria dysfunction. We discuss these mechanisms within the broad scenario of cardiac involvement in sepsis, presenting also our related data obtained on* postmortem* cardiac samples of septic patients.

## 2. NADPH Oxidase-Derived ROS

Oxidative stress (OS) arises as a result of an imbalance between free radical production and antioxidant defense. When antioxidant strategies are overwhelmed, OS results and excessive ROS and reactive nitrosative species (RNS) are produced. ROS can cause oxidation damage to all cellular components, including lipids, proteins, and DNA. The latter is the most detrimental, since replication of damaged DNA can lead to genetic mutations or apoptosis [22–26].

Some of these species interfere with signaling cascades, while others provoke deleterious effects on various biological molecules and structures. It is clear that the increased production of signaling species and strong oxidants act in synergy with collapse in energy metabolism to provoke cell dysfunction, which may result in organ failure and death.

OS in patients with sepsis has been widely described over the last years [27–29], and it is now widely accepted that oxidative stress is central to the etiology of cell and organ dysfunction and tissue damage in sepsis [30–33].

**Figure 1:**
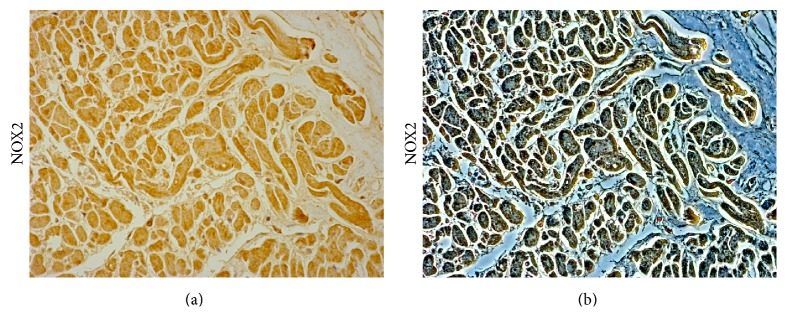
NOX2 expression in the cardiac tissue of a patient died following sepsis. Bright field (a) and contrast phase (b) microscopy image of human cardiac sample, demonstrating a moderate immunopositivity to anti-NOX 2 antibody (Santa Cruz, CA, USA). Personal observation of a 45-year-old septic patient admitted to an emergency unit due to acute abdominal pain and constipation. Abdominal X-ray revealed large amount of fecal content in the colon and dilated small bowel suggestive of bowel obstruction from fecal impaction. The patient was started on broad spectrum antibiotics after pan cultures were obtained and intravenous fluids were administered, and surgical evaluation was requested for possible surgical intervention of bowel obstruction. However, he had a rapid decline in clinical status with worsening hypotension. An echocardiogram revealed severe left ventricular dysfunction (EF 37%) and dilatation of the right ventricle with medium-apical akinesis. Despite a vigorous fluid resuscitation, dopamine, dobutamine, and norepinephrine infusion, the patient died.

Although several sources of ROS may be involved, a family of the NADPH oxidases appears to be especially important for redox signaling; during sepsis, a major source of ROS is the NADPH oxidases that are present in a variety of cells, especially the professional phagocytes and endothelial cells, and that are central to the genesis of the inflammatory response [34] (Figure 1). ROS production after LPS stimulation in leukocytes is primarily mediated by NADPH oxidase activation [35].

NADPH oxidase is a superoxide-generating enzyme comprising a membrane-bound catalytic subunit (NOX) and several cytosolic regulatory subunits. NOX2 is the catalytic subunit of phagocyte NADPH oxidase [35, 36]. On activation, the cytosolic components translocate to the transmembrane catalytic protein gp91^phox^, which results in the formation of functional NADPH oxidase complex. NADPH oxidase expression has been demonstrated in cardiomyocytes [37, 38]. Conclusively, NADPH oxidase is a pivotal source of ROS that subsequently triggers the release of ROS by other enzymes [39], thus playing a pivotal determinant role of the redox state of the myocardium [37, 38, 40, 41], and it has also been implicated in the TNF-*α* production induced by LPS [42].

Experimental data derived from animal models showed a strong increase in NADPH oxidase activity and O_2_^−^ in the heart in response to LPS (lipopolysaccharides) [43, 44]. However, the role of this enzyme complex in myocardial septic depression is not completely clarified, partially due to the fact that the membrane subunit of the NADPH oxidase gp91^phox^ (NOX2) has at least 3 other homologs, NOX1, NOX3, and NOX4 [45], which are expressed in a cell- and tissue-specific fashion, are subject to independent activation and regulation, and may subserve distinct functions [46]. Both gp91^phox^ and NOX4 are expressed in cardiomyocytes [47].

NOX2-derived ROS were likely to mediate hyperinflammatory responses and sepsis-induced mortality in mice [48]. In particular, Peng et al. [42] demonstrated that the subunit gp91^phox^ of NADPH oxidase plays a critical role in myocardial depression induced by endotoxemia and that gp91^phox^-containing NADPH oxidase signaling contributes to LPS-stimulated TNF-*α* expression in cardiomyocytes. Also the role of NOX1/NADPH oxidase in septic myocardial dysfunction has been investigated, and recently Matsuno et al. [49] investigated the involvement of NOX1-derived ROS in endotoxemia-induced cardiac dysfunction. Using an animal model, the authors demonstrated a correlation between the increase of NOX1 mRNA and the production of ROS in cardiac tissue of septic mice and that the increase in cardiomyocytes apoptosis and activation of caspase-3 induced by LPS were attenuated in mice deficient in NOX1. In particular, ROS derived from NADPH oxidase are known to induce cardiomyocytes apoptosis [50], and it has been suggested that NOX1-induced ROS might cause apoptosis by reducing Akt signaling in the heart [49]. Apoptosis may be directly involved in cardiac dysfunction in sepsis as demonstrated in studies on animal models [51], showing that endotoxin may induce a TNF-alpha-dependent apoptotic cascade in the myocardium [52].

Finally, it is noteworthy that complex interactions among different ROS sources exist and that NADPH oxidase-produced ROS may induce ROS production by other sources, thus increasing the total level of ROS [46]. Mitochondrial ROS production can be increased by ROS produced by different sources [53] and it was demonstrated that mitochondrial ROS production may, in turn, stimulate NADPH oxidase ROS production in endothelial cells [54].

## 3. The Role of Mitochondria

Mitochondria, which occupy 30–50% of the cardiomyocyte cytoplasmic volume, are critical in cardiac energy balance since energy supply for cardiomyocytes is mostly derived from mitochondrial oxidative phosphorylation (OXPHOS). On the other hand, they are a favored site of intracellular damage [55–57]. Mitochondrial dysfunction, reflected in the structure, function, and number of mitochondria within the cardiomyocyte, leads to diminished energy production, loss of myocyte contractility, altered electrical properties, and eventual cardiomyocyte cell death [58].

Furthermore, since 1966 when Jensen was among the first investigators to demonstrate that mitochondria produce ROS [59], a solid evidence exists regarding ROS production in mitochondria [60–64]. Mitochondrial dysfunction and its consequence, oxidative stress, have long been considered contributory factors in cardiac tissue damage [65–68].

In sepsis, mitochondrial dysfunction exists [69–72] and mitochondrial damage is thought to play a pivotal role in cardiac dysfunction during sepsis [73, 74]. Several animal models of sepsis have demonstrated cardiac mitochondrial dysfunction during sepsis [75–82]. Evidence exists that mitochondrial dysfunction is a key feature in endotoxemia and the associated multiorgan failure syndrome including heart failure [83]. Soriano et al. [84] studied twenty-five patients presenting with severe sepsis or septic shock and histologically demonstrated on heart sections derangements of mitochondrial cristae in patients who died. Also Takasu et al. found mitochondrial abnormalities in patients who died from sepsis: hydropic change (edema of the mitochondrial matrix), cystic alterations of the cristae, and collapse into small myelin-like clusters were described in septic patients who died in surgical and medical intensive care units [85].

Conclusively, a large body of evidence supports the hypothesis that mitochondrial dysfunction and mitochondria-induced ROS are key factors in cardiac impairment in sepsis [21].

## 4. Nitric Oxide and Peroxynitrite

NO is a free gaseous radical with function of messenger and effector molecule, synthesized by a family of enzymes (nitric oxide synthase (NOS)) [86]. NO synthesis is activated by one of the three isoforms of NOS that are obligated homodimers that catalyze NADPH-dependent oxidation of l-arginine to NO and l-citrulline: NOS1 (neuronal or nNOS), NOS2 (inducible or iNOS), and NOS3 (endothelial or eNOS) [87]. NO is an important bioactive substance which plays an important role in the regulation of normal body function and disease occurrence, and it is recognized to be a ubiquitous signaling molecule with a multitude of biological actions and targets. Signaling may involve direct reactions between NO and a molecular target or can occur through indirect reactions of secondary ROS [88]. In fact, actions of NO are multifaceted, and its interactions with oxygen or oxygen-related reactive intermediates (e.g., superoxide) yield numerous reactive nitrogen as well as oxygen species. These account for most of the so-called indirect effects attributed to NO through oxidation, nitrosation, and nitrate reactions referred to as oxidative, nitrosative, and nitrative stress, respectively. However, much about NO biological actions remains contradictory, especially with regard to pathophysiologic disturbances in NO signaling. There is an ongoing debate about the levels of NO involved, whether there is a clearly defined threshold at which NO crosses from being beneficial to being destructive. Some authors hypothesized that the biological function of NO depends mostly on concentration and time course of exposure to NO, supposing that cytotoxic events, such as arrest of the cell cycle, cell senescence, or apoptosis, can occur at high NO concentrations [89]. However, other authors suggested that the chemical and biological reactivity of NO that has been studied using very high NO concentrations is of doubtful physiological relevance [90].

Vascular bioavailability of NO is a critical factor in regulation of many physiological processes including blood pressure [91], vascular tone [92–95], vascular permeability [96], adhesion and aggregation of platelets [97], and smooth muscle cell proliferation [98]. NO influences on several aspects of cardiomyocytes functioning. The physiological production of NO in the heart maintains coronary vasodilator tone and inhibits platelet aggregation and neutrophil and platelet adhesion, so performing an active role in cardioprotection. It is now determined that NO protects the heart against ischemia-reperfusion injury [99, 100]; however, excessive NO formation is thought to contribute to contractile dysfunction [101, 102].

The inducible NOS (iNOS/NOS2) is generally believed to be the high-capacity NO-producing enzyme in sepsis since endotoxin and cytokines and various mediators are demonstrated to overstimulate the iNOS which is inactive under physiological conditions. Unlike the other two NOS isoforms, iNOS is not constitutively expressed in cells, and its production is elicited by several stimuli like bacterial lipopolysaccharide and cytokines. Primarly identified in macrophages, this enzyme may be expressed in virtually any cell or tissue, such as in the myocardium [103], and once expressed, iNOS is constantly active [87].

**Figure 2:**
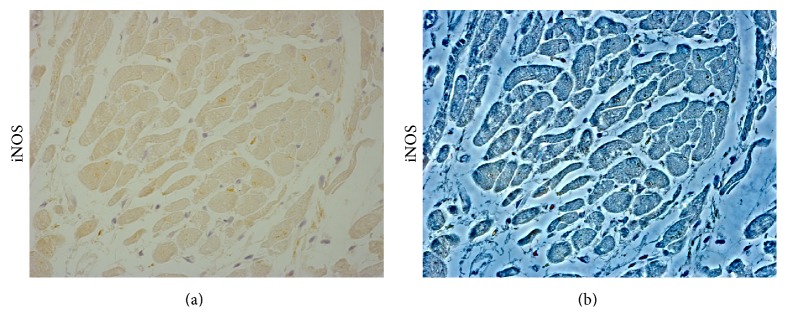
Inducible nitric oxide synthase (iNOS) expression in the cardiac tissue of patient died following sepsis. Bright field (a) and contrast phase (b) microscopy image of cardiac sample, showing a moderate immunopositivity to anti-iNOS antibody (Santa Cruz, CA, USA). Personal observation of a 38-year-old woman with no history of cardiac disease who was referred to an intensive care unit from the obstetrics department 3 days after a cesarean delivery. She was febrile, and laboratory findings included a white blood cell count of 12,800/mm^3^. A postpartum sepsis was diagnosed. Echocardiography showed severe LV systolic function and hypokinesia. Coronary angiography on the same day revealed no significant coronary stenoses. Because of the patient's hemodynamic instability, medical treatment that included inotropic agents and antibiotics was started. She did not respond to the treatment and three days later died.

Although some studies demonstrated that high levels of NO in sepsis could be beneficial due to a bactericidal effect [104], excessive production of NO is an important player during hypotension and catecholamine-resistant septic shock [105] and contributes to myocardial dysfunction [106–109] (Figure 2).

In the inflammatory condition that is central in endotoxemia, NO and peroxynitrite (ONOO^−^) have a central role in the development of mitochondrial dysfunction. Several experimental studies performed on animals subjected to endotoxemia demonstrated that NO production, production of O_2_^∙−^ and H_2_O_2_, global protein nitration, nitrotyrosine content, protein carbonylation, and lipid peroxidation are increased in cardiac mitochondria [76, 80, 81, 110, 111]. Moreover, the antioxidant systems seem to be inhibited as shown by decreased activity of Mn-superoxide dismutase and glutathione peroxidase and depletion of glutathione [112, 113]. In their elegant experiment, Escames et al. [114] demonstrated that increased oxidative stress, impairment in OXPHOS function, and a decrease in ATP production were restored by genetic deletion of iNOS (iNOS^−/−^ mice). This argument is further supported by the fact that treatment with melatonin, an inhibitor of iNOS, prevented the impairment of mitochondrial homeostasis after sepsis, restored ATP production, and improved survival [114]. Other experimental studies conducted in septic animal models demonstrated an improvement of cardiac function by pharmacological inhibition or genetic deletion of NOS [115].

Finally, the possible role of eNOS in cardiac involvement during sepsis has to be discussed. NOS 3 is expressed in endothelial cells and in cardiac myocytes; NOS3-derived NO physiologically has a positive inotropic and lusitropic effect, thus contributing to optimal cardiac performance and filling [116, 117]. Studies demonstrated that during sepsis an increase in iNOS is induced by endotoxins and cytokines while a decrease in eNOS activity would be likely to happen [118]. Functioning of NOS3 in sepsis is not yet completely clarified and controversial results are reported with studies showing that NOS3 has no proinflammatory or anti-inflammatory effects in sepsis [119]. For example, Yamashita et al. examined the effect of chronic eNOS overexpression and the role of eNOS-derived NO in LPS-induced septic shock using eNOS transgenic mice and demonstrated that chronic eNOS overexpression in the endothelium of mice resulted in resistance to LPS-induced hypotension, lung injury, and death. These effects are associated with the reduced vascular reactivity to NO and the reduced anti-inflammatory effects of NO [120]. Other studies postulated a proinflammatory role for eNOS and that eNOS-derived NO is critical for maximal iNOS expression in the vasculature [121, 122]. More recently, Bougaki et al. examined the impact of NOS3 deficiency on systemic inflammation and myocardial dysfunction in vivo and in cardiomyocytes isolated from mice subjected to peritonitis-induced polymicrobial sepsis and reported that NOS3 protects against systemic inflammation and myocardial dysfunction during polymicrobial sepsis [119]. The detrimental effects of NOS3 deficiency on myocardial function appeared to be caused by impaired Ca2+ handling of isolated cardiomyocytes obtained from mice subjected to colon ascendens stent peritonitis. Depressed Ca2+ handling of cardiomyocytes of NOS3KO mice were associated with impairment of mitochondrial integrity and marked depression of the ability of mitochondria to produced ATP, a determinant of the function of the SR Ca2+-ATPase pump [119]. Moreover, Ichinose et al. demonstrated that cardiomyocyte-specific overexpression of eNOS prevented cardiac impairment and death after sepsis induction in mice, thus underscoring an important protective role of myocardial NOS3 against endotoxin-induced myocardial dysfunction and death [123]. Finally, a recent study by van de Sandt et al. demonstrated that endothelial NOS plays a key role in the development of sepsis. In their experiment on male NOS3^−/−^ and C57BL/6 wildtype mice rendered septic by cecum ligation and puncture, the authors found that NOS3 promoted a drop in mean arterial blood pressure and systemic vascular resistance, a hyperdinamc state despite impaired left ventricular function, a rapid deterioration of cardiac output, and limited coronary flow reserve, thus leading to short survival times. These findings were not observed in septic NOS3^−/−^ mice which showed that survival times extended more than twofold [110].

Although a two-step oxidation of l-arginine to l-citrulline, with concomitant production of NO, represents the reaction assumed to be catalyzed by NOS, these enzymes are also capable of catalyzing the production of additional products, notably superoxide anion (O_2_^∙−^), depending on the conditions [124]. During the reaction of molecular oxygen with the amino acid substrate l-arginine to produce l-citrulline and NO, electrons donated by NADPH at the carboxy-terminal reductase domain of NOS are passed to the heme catalytic center of the oxidase domain, where activation of molecular oxygen is “coupled” to NO synthesis by two successive monooxygenations of l-arginine [125]. The cofactor 6R-5,6,7,8-tetrahydrobiopterin (BH4) is required for these reactions; in its absence, electron flow to molecular oxygen becomes “uncoupled” from l-arginine oxidation, resulting in production of O_2_^−^ instead of NO [126, 127]. Furthermore, low concentrations or absence of l-arginine and accumulation of endogenous methylarginines are supposed to cause the uncoupled reduction of molecular oxygen [87]. Both eNOS and iNOS are thought to be involved in the uncoupled reduction of oxygen, leading to the production of O_2_^∙−^ and H_2_O_2_ [124]. These two products can react together extremely rapidly to form the potent oxidant peroxynitrite. Superoxide derived from uncoupled NOS or other mechanisms is an important source of cellular oxidative stress, including BH4 oxidation which may occur both directly by superoxide [128] and through the oxidization to BH2 by ONOO^−^ [129, 130].

Conclusively, NOS uncoupling leads to reduced de novo NO production; sequestration of bioactive NO by superoxide anions via peroxynitrite formation; and peroxynitrite-mediated oxidation of BH4 to BH2, resulting in further propagation of NOS uncoupling [126].

The production of ONOO^−^ is a crucial pathophysiological event which occurs during sepsis since it represents a critical cytotoxic factor in oxidative stress-mediated tissue damage, supposed to be the NO toxicity mediator [131–134], which, in turn, exhibits multiple inhibitory actions in the mitochondrial respiratory chain [135].

**Figure 3:**
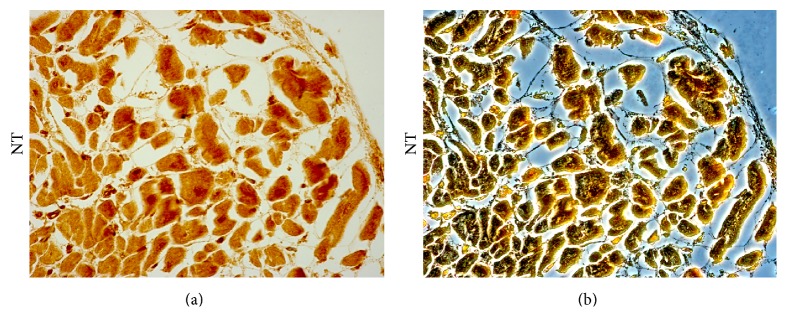
Nitrotyrosine (NT) expression in the cardiac tissue of patient died following sepsis. Bright field (a) and contrast phase (b) microscopy image of cardiac sample of a septic patient, showing a strong immunopositivity to anti-nitrotyrosine antibody (Abcam, Cambridge, UK). Personal observation of a 60-year-old man diagnosed with acute suppurative cholangitis and treated with antibiotic therapy and endoscopic drainage. Jaundice slightly improved; however, on the third day after admission, an echocardiography showed biventricular wall dysfunction. The patient died on third day despite massive fluid and vasopressor support.

ONOO^−^ and its derivatives are able to enter the cell membrane and consequently oxidize multiple target molecules, either directly or through the generation of highly reactive radicals, resulting in structural modification and dysfunctions in lipids, proteins, and nucleic acids with significant cytotoxic consequences [136–139]. ONOO^−^ can also react with carbon dioxide (in equilibrium with physiological levels of bicarbonate anion) leading to the formation of carbonate (CO_3_^∙−^) and nitrogen dioxide (∙NO_2_) radicals, which are one-electron oxidants. ∙NO_2_ can undergo diffusion-controlled radical-radical termination reactions, resulting in nitrated species, such as nitrotyrosine (Figure 3).

**Figure 4:**
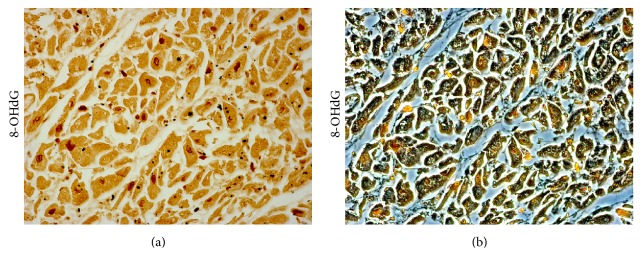
8-Hydroxy-2′-deoxyguanosine (8-OHdG) expression in the cardiac tissue of patient died following sepsis. Bright field (a) and contrast phase (b) microscopy image of cardiac sample of a septic patient, showing a high positive rate of 8-OHdG expression in myocardial nuclei using an anti-8-hydroxy-2′-deoxyguanosine (8-OHdG) antibody (JaICA, Japan). Personal observation of a 31-year-old woman with severe burn wounds of the right thigh. After two days of antibiotic therapy, her clinical condition worsened with the onset of cardiac failure symptoms. Immediate transthoracic echocardiogram revealed profound diffuse hypokinesis and severely depressed systolic function of the left ventricle with ejection fraction of 21%, right ventricular dilatation, and no valvular abnormalities. Fluid and vasopressor support were started; however, several hours later, LV function started to deteriorate with severe hemodynamic instability and progression to death.

Alternatively, it can undergo homolytic fission to generate one-electron oxidants hydroxyl (∙OH) and ∙NO_2_ radicals. The proton-catalyzed decomposition to form ∙OH and ∙NO_2_ radicals may become relevant in hydrophobic phases resulting in the initiation of lipid peroxidation processes [140, 141]. Another important interaction of ONOO^−^ occurs with nucleic acids, with the production of 8-hydroxydeoxyguanosine [142] or 8-nitroguanine [143] (Figure 4).

ONOO^−^ can disrupt DNA integrity, impair the activity of ion channel, break down mitochondrial respiratory chain, and induce cell death. It mediates nitration of tyrosine and cysteine residues in proteins which is one of the crucial pathways contributing to its cytotoxicity [144]. The involvement of ONOO^−^ in sepsis has been demonstrated in different animal models. Several studies suggested that ONOO^−^ is responsible, at least in part, for the development of endotoxin-induced hypotension, endothelial injury, multiple organ dysfunction, and subsequent death [145, 146]. In their nice experiment in sepsis model mice induced by LPS, Okazaki et al. [147] found that the LPS-treated mice were under oxidative stress and that species, such as superoxide and peroxynitrite, were mainly involved in the oxidative stress. In support of these results, superoxide, nitric oxide, and peroxynitrite cardiac formation has been demonstrated in septic hearts, which has been implicated in the pathogenesis of the myocardial depression and cell death in sepsis [148, 149]. Finally, data from the literature showed that ONOO^−^ could mimic many of the cardiovascular alterations associated with shock (endothelial dysfunction, vascular hyporeactivity, myocardial impairment, and cellular energetic failure [150]) and that peroxynitrite neutralizers reduced peroxynitrite accumulation and improved myocardial contractile dysfunction and inflammation in septic animal models [136, 151]. Peroxynitrite contributes to the cardiovascular collapse of septic shock, promoting vascular contractile failure and endothelial and myocardial dysfunction, and is also implicated in the occurrence of multiple organ dysfunction in this setting [152–154].

## 5. Conclusion

Collectively, these findings broaden our understanding of the role of oxidative and nitrosative stress unbalance in the pathophysiology of cardiac dysfunction in sepsis, thus contributing to establishment of a relationship between these settings and the occurrence of oxidative stress. Cardiac impairment is common in severe sepsis and septic shock and it is demonstrated that it strongly contributes to the high rate of mortality in these subjects. Oxidative-nitrosative stress may contribute to cardiac dysfunction in sepsis, and mitochondria are one of the major sites for generation of ROS/RNS as a detrimental side product of oxidative energy metabolism. In turn, damaged mitochondria may increase the production of ROS/RNS.

To improve mortality and morbidity in the septic patient, the most important focus are a prompt and specific management of the infectious finalized to eradicate the causative pathogen as well as supportive therapy to maintain and restore organ function. This strategy is also recognized as the standard therapy for sepsis-induced cardiomyopathy [155]; however, attempts to reduce high mortality rates of patients with sepsis, severe sepsis, and septic shock by manipulating these functional alterations have provided limited success [156]. The complex pathogenesis of septic cardiac failure, involving a combination of interconnected hemodynamic, structural, metabolic, molecular, and genetic alterations, may explain why despite all these therapeutic strategies, sepsis remains a big clinical challenge for effectively managing the disease to minimize mortality [157]. Numerous basic research and clinical trials have been undertaken to evaluate the possible modulation of the uncontrolled response in sepsis [158]. The knowledge that unbalanced oxidative stress could be critical in the pathophysiology of cardiac impairment in sepsis has naturally led to consideration of the potential therapeutic effects of antioxidant agents [159]. Over the years, treatment of the impaired myocardial energetics rather than cardiac inflammation has been postulated to curb the lethal tools of sepsis with drugs that target the oxidative stress unbalance and the deep mechanisms within mitochondria [21, 160–167].
